# Fraxetin Suppresses Cell Proliferation and Induces Apoptosis through Mitochondria Dysfunction in Human Hepatocellular Carcinoma Cell Lines Huh7 and Hep3B

**DOI:** 10.3390/pharmaceutics13010112

**Published:** 2021-01-17

**Authors:** Jisoo Song, Jiyeon Ham, Taeyeon Hong, Gwonhwa Song, Whasun Lim

**Affiliations:** 1 Department of Food and Nutrition, College of Science and Technology, Kookmin University, Seoul 02707, Korea; js_song97@kookmin.ac.kr (J.S.); taeyeon97@kookmin.ac.kr (T.H.); 2 Institute of Animal Molecular Biotechnology, Department of Biotechnology, College of Life Sciences and Biotechnology, Korea University, Seoul 02841, Korea; gloryjy76@korea.ac.kr

**Keywords:** fraxetin, hepatocellular carcinoma, mitochondria, signal transduction

## Abstract

Fraxetin is a coumarin scaffold compound extracted from *Fraxinus rhynchophylla*. It has antioxidant, anti-inflammatory, hepatoprotective, and antifibrotic effects. Furthermore, fraxetin has anticancer effects in breast and lung cancer. We aimed to evaluate whether fraxetin has anticancer activity in hepatocellular carcinoma (HCC) cells and its underlying mechanism. We demonstrated the anticancer effects of fraxetin in the HCC cell lines Huh7 and Hep3B. We confirmed that fraxetin inhibited cell proliferation (42% ± 10% Huh7; 52% ± 7% Hep3B) by arresting the cell cycle and inducing apoptosis in both cell lines. Moreover, fraxetin increased reactive oxygen species production (221% ± 55% Huh7; 460% ± 73% Hep3B), depolarized the mitochondrial membranes (ΔΨm) (345% ± 160% Huh7; 462% ± 140% Hep3B), and disrupted calcium homeostasis in both HCC cell lines. Chelating calcium ions with BAPTA-AM restored proliferation in fraxetin-treated Huh7 cells but not in Hep3B cells. Fraxetin did not affect the phosphorylation of extracellular-signal-regulated kinase 1/2, whereas it decreased JNK and phosphoinositide 3-kinase signaling. Furthermore, fraxetin and mitogen-activated protein kinase pharmacological inhibitors had synergistic antiproliferative effects on HCC cells. Although our study was limited to in vitro data that require validation, we suggest that fraxetin is a potential therapeutic agent against HCC progression.

## 1. Introduction

Hepatocellular carcinoma (HCC) was the fifth (among males) and seventh (among females) most lethal cancer worldwide in 2020 and only 18% of HCC patients survive 5 years with the disease [1]. Causes of HCC include infection by hepatitis viruses, development of non-alcoholic fatty liver diseases or steatohepatitis, and exposure to toxic compounds. Sorafenib is a well-known chemotherapeutic agent for HCC, renal, and thyroid cancers, which was approved by the Food and Drug Administration. Sorafenib blocks multiple kinases, especially Raf, thereby regulating the proliferation and angiogenesis of tumors [2]. However, some tumors acquire chemoresistance against sorafenib, which reduces its efficacy and increases its systemic toxicity [3]. Therefore, new agents and methods to complement the current therapy are being actively researched. To overcome chemoresistance, one strategy is to supplement the standard agents with natural compounds [4,5]. For example, esculetin is a coumarin scaffold compound that induces mitochondrial apoptosis and suppresses the proliferation of HCC cells and mouse liver cancer models [6].

Coumarin scaffold compounds have anti-inflammatory, antioxidant, hepatoprotective, and anticarcinogenic effects [7]. Fraxetin is a hydroxycoumarin compound isolated from the stem or root bark of *Fraxinus rhynchophylla*, a traditional medicinal plant [8,9,10,11]. Fraxetin exhibits anticancer activity in breast cancer by regulating pro/anti-apoptotic protein expression [11] and antifibrotic activity in carbon tetrachloride4-induced liver fibrosis in rats by regulating inflammatory pathways [12]. Given its potential ability to eliminate cancer cells, the role of fraxetin and its underlying mechanisms in HCC are worthy of study. The development of new anticancer agents requires identification of new molecules and characterization of their activities and underlying mechanisms before further drug development. We explored the anticancer capacities and intracellular mechanisms of fraxetin in HCC cell culture.

Therefore, we investigated the ability of fraxetin to (1) inhibit cellular proliferation, (2) induce cell cycle arrest and apoptosis, (3) hamper mitochondrial function and calcium homeostasis, (4) regulate gene transcription, and (5) dysregulate intracellular signaling pathways, mitogen activated protein kinase (MAPK) and phosphoinositide 3-kinase (PI3K), in HCC. Our results suggest that fraxetin is a potentially useful chemotherapeutic agent or supplementation for HCC.

## 2. Materials and Methods

### 2.1. Reagents

Fraxetin (Cat No. 18224) was purchased from Sigma-Aldrich (St. Louis, MO, USA) and dissolved in dimethyl-sulfoxide (DMSO). The antibodies against phosphorylated extracellular-signal-regulated kinase (ERK)1/2 (Thr^202^/Tyr^204^, Cat No. 9101), JNK (Thr^183^/Tyr^185^, Cat No. 4668), P90RSK (Thr^573^, Cat No. 9346), P70S6K (Thr^421^/Ser^424^, Cat No. 9204), S6 (Ser^235^/^236^, Cat No. 2211) and the total form of ERK1/2 (Cat No. 4695), JNK (Cat No. 9252), RSK1/RSK2/RSK3 (Cat No. 9355), P70S6K (Cat No. 9202), and S6 (Cat NO. 2217) were purchased from Cell Signaling Technology (Beverly, MA, USA).

### 2.2. Cell Culture

The HCC-derived cell lines Huh7 and Hep3B were purchased from Korea Cell Line Bank (KCLB, Seoul, Korea) and cultured following the KCLB cell culture guidelines (https://cellbank.snu.ac.kr). The cell culture medium was RPMI-1640 with 25 mM HEPES and l-Glutamine medium for Huh7 cells and DMEM/High glucose medium with 10% fetal bovine serum (FBS) for Hep3B cells. The cells were incubated in 100 mm cell culture dishes until they reached 70% confluence and were treated with different concentrations of fraxetin with or without inhibitors for 48 h.

### 2.3. Cell Proliferation Measurements

Proliferation assays were performed using the Cell Proliferation ELISA, BrdU kit (Cat No. 11647229001; Roche, Basel, Switzerland) according to the manufacturer’s manual as described previously [13]. Briefly, both Huh7 and Hep3B cells were cultured in 96-well plates and starved for 24 h in serum-free medium. HCC cells were incubated with various doses of fraxetin with or without BAPTA-AM (calcium chelator), U0126 (ERK1/2 inhibitor), or SP600125 (JNK inhibitor) in a maximum volume of 100 μL/well for 48 h. After BrdU labeling, the cells were fixed and anti-BrdU-POD was added. After 90 min of incubation, the absorbance was measured at wavelengths of 370 nm and 420 nm using a microplate spectrophotometer.

### 2.4. Immunofluorescence Detection of Proliferative Cell Nuclear Antigen (PCNA)

The effect of fraxetin on the expression level of PCNA was determined using immunofluorescence microscopy. In brief, cells were seeded in confocal dishes and starved for 24 h in serum-free medium. Next, the cells were incubated with fraxetin (20 μM for Huh7 or 50 μM for Hep3B) for 48 h at 37 °C in a 5% CO_2_ incubator. After treatment, the cells were blocked with goat serum and stained using PCNA. Subsequently, the secondary antibody for PCNA and 4′,6′-diamidino-2-phenylinodole (DAPI, Cat No. D8417; Sigma-Aldrich) were added. The fluorescence of the confocal dishes was measured using a confocal microscope (LSM710, Carl Zeiss, Oberkochen, Germany). A previous study described this process in detail [14].

### 2.5. Cell Cycle Progression Analysis

The changes in the cell cycle stage by fraxetin were assessed using propidium iodide (PI). Briefly, the Huh7 and Hep3B cells were seeded in 6-well plates and starved. They were then incubated with fraxetin (0, 5, 10, and 20 or 0, 10, 20, and 50 μM) for 48 h at 37 °C in a 5% CO_2_ incubator. The cells were fixed with 0.1% BSA PBS and chilled in 70% ethanol at 4 °C overnight. The cells were treated with 10 mg/mL RNase A (Sigma-Aldrich) and 50 mg/mL PI (BD Biosciences, Franklin Lakes, NJ, USA) and incubated for 30 min at room temperature. The results were measured using fluorescence-activated cell sorting (FACS). This assay was performed following a previous study [13].

### 2.6. Detection of Apoptotic Cells

Apoptosis induced by fraxetin in Huh7 and Hep3B cells was analyzed using the FITC Annexin V apoptosis detection kit I (BD Biosciences) according to the manufacturer’s manual. After collection, the fraxetin-treated cells were washed with PBS several times and then stained with Annexin V and PI at room temperature. FACS were used to detect the stained cells and the results were analyzed using CellQuest software. This assay was performed following a previous study [14].

### 2.7. Mitochondrial Membrane Potential (MMP) Assay

Changes in MMP by fraxetin in Huh7 and Hep3B cells were detected using a mitochondrial staining kit (Cat No.: CS0390; Sigma-Aldrich). In accordance with the manufacturer’s manual, prepared cells were stained with JC-1 staining solution and incubated for 20 min at 37 °C incubators. After washing with a staining buffer, JC-1 stained-cells were detected using FACS. This assay was performed following a previous study [14].

### 2.8. Reactive Oxygen Species (ROS) Assay

The level of ROS following fraxetin treatment was monitored using 2′-7′-dichlorofluorescein diacetate (DCFH-DH; Sigma-Aldrich), which was converted to 2′-7′-dichlorofluorescein (DCF) by peroxides. In short, Huh7 and Hep3B cells were treated with 10 μM of DCFH-DH and then washed with 1× PBS. The cells were analyzed using FACS. This assay was performed following a method outlined in a previous study [14].

### 2.9. Real-Time Quantitative Polymerase Chain Reaction (RT-qPCR) Analysis

The cells were incubated with fraxetin for 24 h and then RNA was extracted from each cell line. The expression level of each gene was detected with SYBR Green (Sigma-Aldrich) on a StepOnePlus Real-Time PCR system (Applied Biosystems, Foster City, CA, USA). We measured the expression level of target genes listed in Table 1 using standard curves, CT values, and the expression of *GAPDH*. The relative gene expression was analyzed using the 2-∆∆ CT method [13].

### 2.10. Cytosolic Calcium Ion Concentration Assay

The cytosolic calcium ion level was measured using Fluo-4 AM dye (Invitrogen, Waltham, MA, USA). Simply, fraxetin-treated cells were stained with 3 μM Fluo-4 AM for 20 min and washed with 1× PBS. Next, the cells were analyzed using FACS. This assay was performed following a previous study [14].

### 2.11. Mitochondrial Matrix Calcium Ion Concentration Assay

The mitochondrial calcium ion level was detected using 3 μM Rhod-2 AM (Invitrogen, Carlsbad, CA, USA). Briefly, identical cells were prepared and treated with fraxetin for 48 h; subsequently, the cells were collected and stained using Rhod-2 AM for 30 min. Next, the cells were treated with Hank’s balanced salt solution (HBSS; Gibco), incubated for 10 min, and then analyzed using FACS. This assay was performed following a previous study [14].

### 2.12. Western Blotting

The concentration of the proteins extracted from whole cells was determined using the Bradford assay (Bio-Red, Hercules, CA, USA) with BSA as the standard. The proteins were denatured and isolated via 10% sodium dodecyl sulfate-polyacrylamide gel electrophoresis and then transferred to a nitrocellulose membrane. Primary and secondary antibodies for each protein were added in a serial order and measured using the ChemiDoc EQ system and Quantity One software (Bio-Rad). The assay was performed as described previously [14].

### 2.13. Statistical Analysis

All data were subjected to analysis of variance following the general linear model (PROC-GLM) of the SAS program (SAS, Institute, Cary, NC, USA) to confirm whether there were significant differential effects on Huh7 and Hep3B cells in response to treatments [15]. Differences with a probability value of <0.05 were considered statistically significant. Data are presented as mean ± standard deviation of the mean unless otherwise stated.

## 3. Results

### 3.1. Fraxetin Suppresses the Proliferation of Huh7 and Hep3B Cells

**Figure 1:**
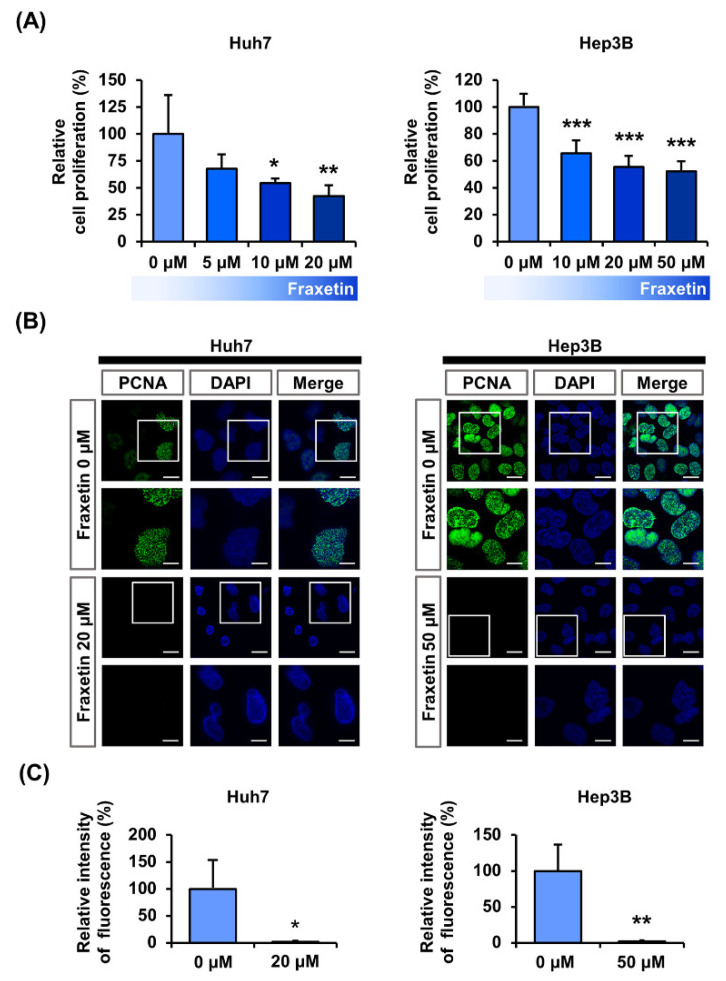
Antiproliferation effects of fraxetin in human hepatocellular carcinoma. (**A**) Cell proliferation of Huh7 and Hep3B in response to various concentration of fraxetin (0, 5, 10, and 20 or 0, 10, 20, and 50 μM). Average values of triplicated data converted to relative ratio values. (**B**) Confocal images of Huh7 and Hep3B cells. Green fluorescence indicates PCNA and blue fluorescence indicates DAPI. DAPI stained nuclei for co-localization. Scale bars: 20 μm. (**C**) Relative fluorescence intensity between vehicle and fraxetin treatment (20 μM or 50 μM). Asterisk marks indicate significant levels between vehicle- and fraxetin-treated cells (* *p* < 0.05, ** *p* < 0.01, and *** *p* < 0.001).

We measured cell viability at different concentrations of fraxetin (0, 5, 10, 20, and 50 μM) in Huh7 and Hep3B cells as shown in Figure 1A. At 20 µM, fraxetin reduced the proliferation of Huh7 cells by >2-fold (*p* < 0.01) and at 50 μM, fraxetin reduced the proliferation of Hep3B cells by two-fold (*p* < 0.001). In addition, fraxetin reduced the relative green fluorescence (indicative of the proliferation marker PCNA) by >90% in Huh7 cell (20 μM, *p* < 0.05) and Hep3B cells (50 μM, *p* < 0.01) compared to vehicle-treated cells (Figure 1B,C). These results show that fraxetin reduced the proliferation of HCC cells.

### 3.2. Fraxetin Induces Cell Cycle Arrest and Apoptosis in Huh7 and Hep3B Cells

**Figure 2:**
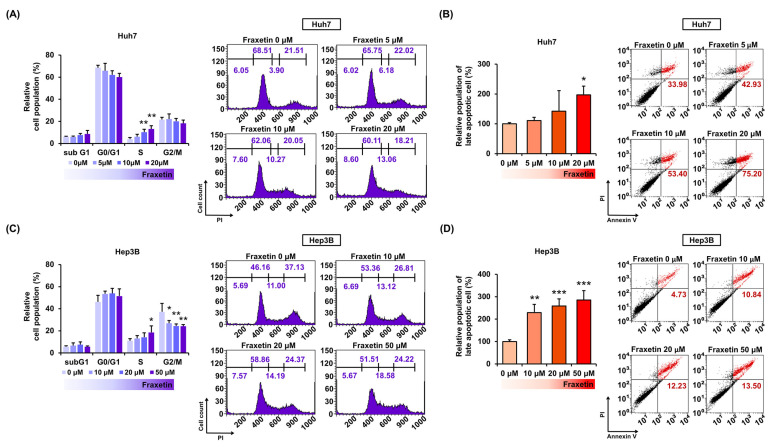
Effects of fraxetin on cell cycle arrest and apoptosis in Huh7 and Hep3B cells. (**A**,**B**) Cell cycle arrest in Huh7 and Hep3B cells was confirmed using propidium iodide (PI) staining and flow cytometry (FACS). (**C**,**D**) The hepatocellular carcinoma cells were stained with annexin V and PI to detect late apoptotic cells via FACS. The late apoptotic cell population are located in the upper right quadrant and the bar graph represents the percentage ratio values. Asterisks indicate the significance levels of comparisons between vehicle- and fraxetin-treated cells (* *p* < 0.05, ** *p* < 0.01, and *** *p* < 0.001).

Next, we investigated the effect of fraxetin on cell cycle arrest in HCC cells using PI staining. Fraxetin treatment (0, 5, 10, 20, and 50 μM) gradually increased the relative population of S phase cells in both cell lines (Figure 2A,B). Moreover, fraxetin gradually decreased the G2/M cell population in Hep3B cells (Figure 2B). Next, we stained fraxetin-treated Huh7 and Hep3B cells with annexin V and PI to investigate apoptosis induction (Figure 2C,D). Fraxetin increased the number of late apoptotic cells in Huh7 and Hep3B in a dose-dependent manner. The late apoptotic cell populations of Huh7 and Hep3B cells increased to 197% (*p* < 0.05) and 285% (*p* < 0.001), respectively, compared to vehicle-treated cells. In short, fraxetin induced cell cycle arrest and apoptosis in Huh7 and Hep3B cells.

### 3.3. Fraxetin Induces a Loss of Mitochondrial Membrane Potential and Increases ROS Production in Huh7 and Hep3B Cells

**Figure 3:**
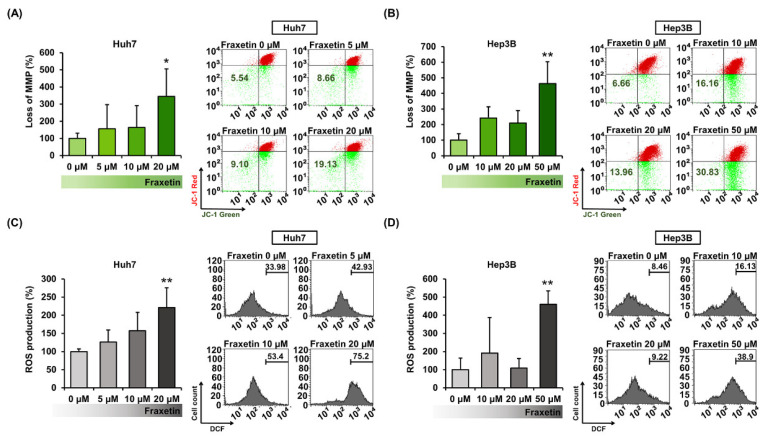
Effects of fraxetin on the mitochondrial function of hepatocellular carcinoma (HCC) cells. (**A**,**B**) Mitochondrial membrane potential (ΔΨm). Huh7 and Hep3B cells. The amounts of cells in the lower right quadrants are represented as a percentage-ratio in the bar graphs. (**C**,**D**) Reactive oxygen species (ROS) in Huh7 and Hep3B cells. The right part of peaks was measured and the values are represented as a percentage-ratio in the bar graphs. Asterisks indicate the significance levels of comparisons between vehicle-treated cells and fraxetin-treated cells (* *p* < 0.05 and ** *p* < 0.01).

We evaluated the effects of fraxetin on mitochondrial function by monitoring the MMP (∆ψ) and the generation of ROS in HCC cells. Fraxetin depolarized MMP in Huh7 and Hep3B cells (Figure 3A,B). At 20 µM in Huh7 cells, fraxetin increased the relative MMP loss ratio by 3.5-fold (*p* < 0.05), whereas at 50 µM in Hep3B cells, it increased by 4.6-fold (*p* < 0.01). Besides, 20 µM of fraxetin increased the production of ROS by 221% in Huh7 cells (*p* < 0.01), while 50 µM increased it by 460% in HEP3B cells (*p* < 0.01) compared to vehicle-treated cells (Figure 3C,D). These results show that fraxetin induces mitochondrial dysfunction and disrupts the oxidative stress-buffering system.

### 3.4. Fraxetin Downregulated the Oxidative Stress-Related Genes in Human HCC Cells

**Figure 4:**
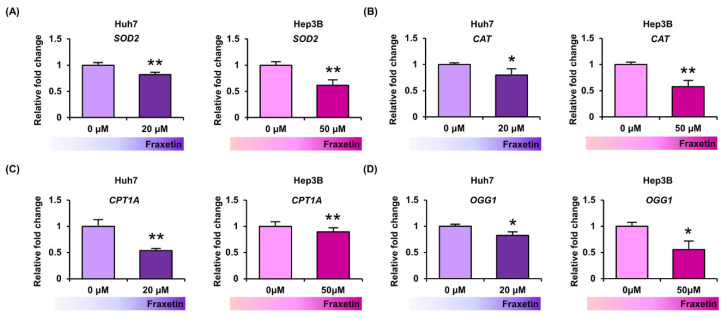
Effects of fraxetin on oxidative stress-related gene expression. (**A**–**D**) Quantitative analysis of mRNA levels of *SOD2* (**A**), *CAT* (**B**), *CPT1A* (**C**), and *OGG1* (**D**) normalized relatively to the house-keeping gene GADPH. RNA was extracted after fraxetin treatment (20 μM or 50 μM) for 24 h on Huh7 and Hep3B cells. Asterisks indicate the significance levels of comparisons between vehicle- and fraxetin-treated cells (* *p* < 0.05 and ** *p* < 0.01).

Next, we confirmed the expression changes of oxidative stress-related genes using quantitative RT-PCR analysis. Fraxetin reduced the mRNA expression of *superoxide dismutase 2* (*SOD2*) to 82% (*p* < 0.01, Huh7) and 61% (*p* < 0.01, Hep3B) compared to the control (100%) (Figure 4A). Fraxetin also decreased the expression of the antioxidant enzyme *catalase* (*CAT*) to 80% (*p* < 0.05) and 58% (*p* < 0.01) in Huh7 and Hep3B cells, respectively (Figure 4B). Finally, fraxetin significantly reduced the expression of *carnitine O-palmitoyltransferase 1* (*CPT1A*) and *oxoguanine glycosylase* (*OGG1*) genes, which are related to fatty acid oxidation and DNA repair process, respectively (Figure 4C,D). Fraxetin produced minor inhibition of oxidative stress-related genes, thus suggesting that it is a potent disruptor of the mitochondrial antioxidant defense system.

### 3.5. Fraxetin Disrupts the Calcium Homeostasis in the Cytoplasmic and Mitochondrial Matrix of HCC Cells

**Figure 5:**
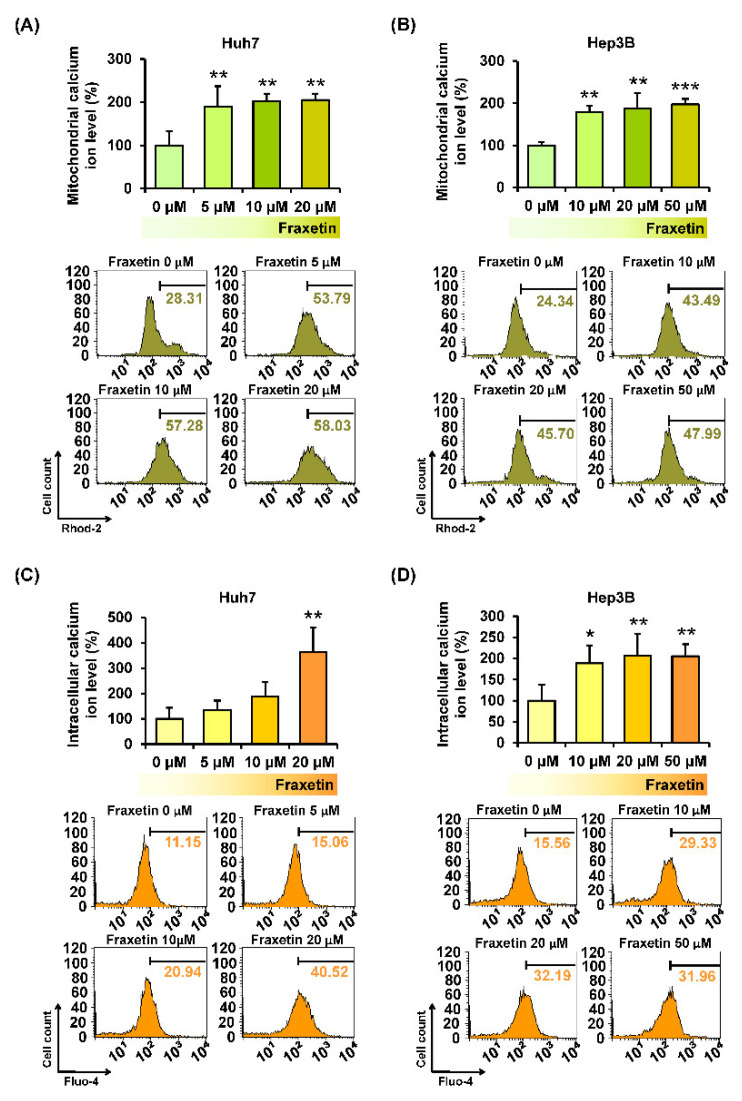
Effects of fraxetin on calcium homeostasis in the mitochondrial matrix and cytoplasm of Huh7 and Hep3B cells. (**A**,**B**) Accumulation of calcium in the mitochondrial matrix. The right part of the peaks were measured and represented as a percentage-ratio in the bar graphs. (**C**,**D**) Intracellular calcium concentrations. Asterisks indicate the significance levels of comparisons between vehicle- and fraxetin-treated cells (* *p* < 0.05, ** *p* < 0.01, and *** *p* < 0.001).

**Figure 6:**
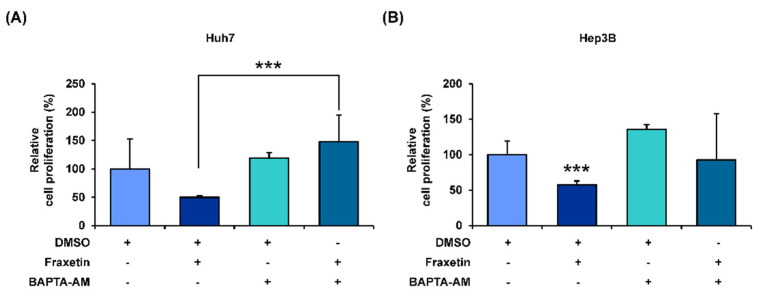
Regulatory effects of calcium ion on the proliferation of human HCC cells. (**A**,**B**) Relative proliferation of Huh7 (**A**) and Hep3B (**B**) cells treated with fraxetin and with or without BAPTA-AM for 48 h. Asterisks indicate the significance levels of comparisons between the vehicle and fraxetin with BAPTA treatments (*** *p* < 0.001).

The disruption of calcium homeostasis in the mitochondria and cytoplasm is related to mitochondria dysfunction. Thus, we stained HCC cells with Rhod-2 and Fluo-4 dye to measure the hampering effect of fraxetin on calcium homeostasis. Intramitochondrial calcium levels ([Ca^2+^]_mito_) in fraxetin-treated Huh7 and Hep3B cells were almost 2-fold higher than in vehicle-treated cells (Figure 5A,B). Besides, fraxetin increased the intracellular calcium levels ([Ca^2+^]_cyto_) of Huh7 up to 363% (*p* < 0.01) compared to vehicle-treated cells (Figure 5C). Fraxetin also increased [Ca^2+^]_mito_ and [Ca^2+^]_cyto_ of Hep3B by 2-fold (Figure 5D). Moreover, we verified whether calcium ion regulation by fraxetin affected the proliferative capacity of HCC by performing a cell proliferation assay with the calcium chelator BAPTA-AM. Chelating the calcium ions in the cytosol by BAPTA-AM treatment restored the fraxetin-reduced proliferation in Huh7 and Hep3B cells (Figure 6A,B). This suggests that the antiproliferation effects of fraxetin on Huh7 and Hep3B cells are mediated by calcium upregulation.

### 3.6. Fraxetin Regulates the Proliferation of HCC Cells via the MAPK and PI3K Pathways

**Figure 7:**
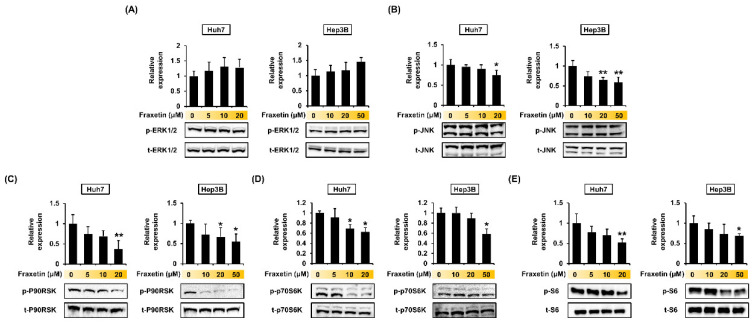
Regulation of mitogen activated protein kinase (MAPK) and phosphoinositide 3-kinase (PI3K) signaling pathways by fraxetin in hepatocellular carcinoma cells. (**A**–**E**) Phosphorylation of ERK1/2 (**A**), JNK (**B**), P90RSK (**C**), P70S6K (**D**) and S6 (**E**) in response to increasing doses of fraxetin. Asterisks indicate the significance levels of comparisons between vehicle-treated cells and fraxetin-treated cells (* *p* < 0.05 and ** *p* < 0.01).

**Figure 8:**
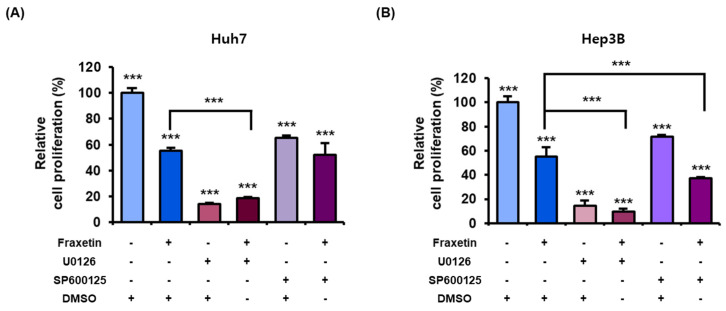
Synergistic effects of fraxetin with MAPK inhibitors on the proliferation of HCC cells. (**A**,**B**) Relative proliferation of cells treated with 20 μM of MAPK inhibitors (U0126: ERK1/2 inhibitor; SP600125: JNK MAPK inhibitor) and fraxetin (20 μM in Huh7 or 50 μM in Hep3B) for 48 h in HCC cells, then each cell proliferation was measured. Asterisks indicate the significance levels of comparisons between the control and treated groups, and fraxetin with or without MAPK inhibitors (*** *p* < 0.001).

Fraxetin treatment (0, 5, 10, and 20 μM for Huh7 and 0, 10, 20, and 50 μM for Hep3B cells) did not significantly affect the phosphor-ERK1/2 protein levels (Figure 7A). However, the phosphorylation levels of JNK, P90RSK, P70S6K, and S6 decreased as the dose of fraxetin increased in both HCC cell lines (Figure 7B–E). To verify whether the effects of fraxetin on the proliferation of HCC cells were mediated by those pathways, we cotreated the cells with pharmacological inhibitors of these proteins and fraxetin for 48 h. U0126 (20 μM, ERK1/2 MAPK inhibitor) with or without fraxetin (20 μM for Huh7 and 50 μM for Hep3B) decreased the proliferation of HCC cells by >5-fold (Figure 8). SP600125 alone (20 μM, JNK MAPK inhibitor) reduced the proliferation to 65% in Huh7 cells and 71% in Hep3B cells, compared to the vehicle. Cotreatment with fraxetin and SP600125 did not have a synergistic effect on the proliferation of Huh7 cells. However, in Huh7 cells, cotreatment with fraxetin and SP600125 decreased the proliferation by approximately 20% compared with SP600125 alone. Furthermore, combining fraxetin with U0126 and SP600125 reduced the proliferation of Huh7 and Hep3B cells to 83% and 33%, respectively, compared with fraxetin alone. Moreover, cotreating Hep3B cells with fraxetin and U0126 decreased the proliferation to 35% compared with U0126 alone. Similarly, cotreating Hep3B cells with fraxetin and SP600125 reduced the proliferation to 48% compared with SP600125 alone. These results indicate that fraxetin inhibits HCC cell proliferation by regulating MAPK signal transduction, although some cell lines differ in their response to pharmacological inhibitors.

## 4. Discussion

**Figure 9:**
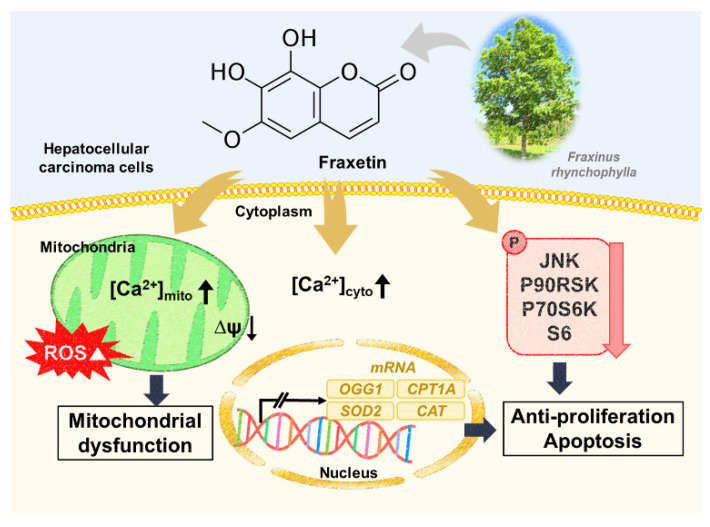
Schematic illustration of fraxetin in HCC cells.

In this study, we demonstrated the anticancer effect of fraxetin on human HCC cell lines. Although previous studies have shown the anticancer effects of fraxetin on various types of cancer cells, this is the first study that has shown its effects on HCC cells. In non-small-cell lung cancer, fraxetin displayed antiproliferation effects and induced cell cycle arrest by blocking STAT3 [16]. Furthermore, fraxetin suppressed the proliferation of human breast cancer cells (MCF-7) by activating the mitochondrial-apoptosis signaling pathway with chromatin condensation [11]. We showed that fraxetin had antiproliferation effects in Huh7 and Hep3B cells by reducing PCNA expression and arresting the cell cycle, which is consistent with previous studies. In addition, we showed that fraxetin increased the late apoptotic cell population in HCC cells. Thus, the present study demonstrated the inhibitory effects of fraxetin on HCC cells (Figure 9).

Changes in calcium ion levels in cell organelles are related to mitochondrial dysfunction and can cause apoptosis. For instance, intracellular calcium ion upregulation in human hepatoma cells is usually associated with oxidative stress and mitochondrial dysfunction, leading to antiproliferation and cell death [17]. The calcium levels in both the cytosol and mitochondrial matrix are involved in the progression of cancers including HCC [18,19]. A calcium overload in the mitochondria initiates mitochondria-mediated apoptosis pathway. The common chemotherapeutic agents for HCC, sorafenib and ascorbate, have synergistic cytotoxic effects and disrupt calcium homeostasis by accumulating massive amounts of calcium in mitochondria [20]. Thus, calcium uptake in mitochondria is a way for anticancer agents to control proliferation and cell death during cancer [21]. In our study, chelating intracellular calcium ion with BAPTA completely restored the fraxetin-reduced proliferation of HCC cells, implying that calcium dysregulation is a key mechanism underlying the antiproliferative effect of fraxetin.

Mitochondria have several important functions to maintain healthy cells. Those mitochondrial functions are affected by the disruption of bioenergetics and the antioxidant defense system, which leads to a calcium imbalance and even apoptosis [22]. First, with regard to bioenergetics, the mitochondria are the primary energy production sites. Maintaining MMP is therefore crucial for ATP production and requires proper mitochondrial calcium uptake [22,23,24]. The opening of the permeability transition pore or other calcium channels can induce mitochondrial calcium accumulation [25,26]. Next, the accumulated calcium in mitochondria disrupts MMP and releases cytochrome c, which initiates mitochondrial apoptosis [27]. For instance, nitric oxide treatment in HCC cells induces the uncoupling of MMP and release of cytochrome c, leading to apoptosis [28]. Second, the mitochondria act as an antioxidant defense system. During mitochondrial respiration, ROS production is inevitable and controlled by several mechanisms. Considering the results of previous studies and those of the present study, fraxetin seemed to have antiproliferative properties and mitochondria-mediated apoptosis effects on HCC cells, because it disrupted MMP, the antioxidant defense system, and calcium homeostasis [11,16,29].

*SOD2* is a mitochondrial matrix enzyme that neutralizes superoxide anions and produces hydrogen peroxide to defend cells against oxidative stress. Furthermore, *CAT* is an antioxidant enzyme that converts hydrogen peroxide to water. Consistent with our results on decreased antioxidant-related genes, ROS generation suppresses *SOD*2 expression via p53 and activates mitochondrial apoptosis in response to pterostilbene (a stilbenoid that is chemically similar to resveratrol) [30]. Furthermore, the *CPT1A* enzyme is responsible for importing Acyl-CoA to mitochondria for fatty acid oxidation [31,32]. The upregulation of *CPT1A* could promote the development of HCC and metastasis of colon cancer [33]. Similarly, *OGG1* contributes to the elimination of the bases damaged by ROS. Downregulation of *OGG1* might sensitize tumor cells to anticancer drugs by accumulating DNA damage [34]. Therefore, the effect of fraxetin on these target genes in HCC cells suggests its involvement in mitochondrial oxidative stress defense and inhibition of cancer development.

MAPKs and PI3K are major regulators of cell survival and apoptosis in human cancers [35]. JNK is a MAPK that might be related to fraxetin-induced cell cycle arrest. Our results indicated that fraxetin affects the cell cycle and proliferation via the MAPK signaling pathway. Similarly, a previous study indicated that curcumin suppresses metastasis and induces the G2/M phase arrest by upregulating the JNK expression in liver cancer [36]. Although fraxetin did not affect the phosphorylation of ERK1/2, it dramatically reduced the expression of P90RSK, which is downstream of ERK1/2. The inhibition of the ERK/P90RSK signal is associated with tumor growth suppression in HCC [37]. Our results on the antiproliferation effects of U0126 with or without fraxetin seem to support the previous studies, even though fraxetin did not affect ERK1/2 phosphorylation. Anticancer agents often downregulate the PI3K/AKT/mTOR pathways because these proteins are involved in various physiological functions such as proliferation, growth, and cell cycle regulation [38,39]. In HCC cells, AKT/mTOR/S6 are involved in lipogenesis and tumor growth. Downregulating those signaling pathways is a therapeutic strategy against liver cancer [40]. In summary, fraxetin showed antiproliferative and apoptotic effects in HCC by inducing mitochondria dysfunction and inactivating the MAPK and PI3K pathways, except ERK 1/2. This is the first study to demonstrate the effects of fraxetin in HCC. In addition, based on a SMILE structure analysis, this study notes that fraxetin can potentially bind to members of the carbonic anhydrase family [41]. The carbonic anhydrase family, especially carbonic anhydrase-IX, is involved in cancer progression because it activates under hypoxic conditions and promotes cancer invasion or metastasis formation [42,43]. Moreover, those enzymes are associated with cancer drug resistance, and they are new targets for various cancers [44]. Our study was performed exclusively in vitro, but despite this limitation, our description of the basic cellular mechanisms underlying a potential anticancer therapeutic is meaningful. Further study and verification may lead to fraxetin serving as a new chemotherapeutic agent for HCC.

## 5. Conclusions

Fraxetin induces human HCC cell death by inducing cell cycle arrest. It also causes the depolarization of mitochondrial membrane potentials, thereby disrupting calcium homeostasis and ROS generation by inhibiting proliferation. Furthermore, fraxetin regulates the JNK and PI3K pathways of mitotic division in HCC cells. Animal studies should be performed to further study this agent for future therapeutic use. Fraxetin should also be combined with sorafenib, the standard therapeutic agent for HCC, to compare the medications and determine whether they are more effective in combination.

## Figures and Tables

**Table 1:** Primer sets used in quantitative RT-PCR.

Gene	Genebank No.	Froward Primer (5′→3′)	Reverse Primer (5′→3′)
*GAPDH*	NM_001289745.3	GGCTCTCCAGAACATCATCC	TTTCTAGACGGCAGGTCAGG
*SOD2*	NM_000636.4	GACAAACCTCAGCCCTAACG	AACCTGAGCCTTGGACACC
*CAT*	NM_001752.4	GGTTCAGCTGACACAGTTCG	CCAACGAGATCCCAGTTACC
*CPT1A*	NM_001031847.3	GATCCACGATTCCACTCTGC	GCTTGCTGTCTCTCATGTGC
*OGG1*	NM_001354649.2	CTCCACTGCACTGTGTACCG	TGAGCCAGGGTAACATCTAGC

## Data Availability

Data is contained within the article.
